# Exergames for health and fitness: the roles of GPS and geosocial apps

**DOI:** 10.1186/1476-072X-12-18

**Published:** 2013-04-05

**Authors:** Maged N Kamel Boulos, Stephen P Yang

**Affiliations:** 1 University of Plymouth, Drake Circus, Plymouth, Devon PL4 8AA, UK; 2 SUNY Cortland, Cortland, NY 13045, USA

**Keywords:** GPS (global positioning system), Geosocial apps, Location-based games, Exergames, Physical activity, Smartphones

## Abstract

Large numbers of children and adolescents in Canada, UK and USA are not getting their recommended daily dose of moderate to vigorous physical activity, and are thus more prone to obesity and its ill health effects. Exergames (video games that require physical activity to play) are rapidly gaining user acceptance, and may have the potential to increase physical activity levels among young people. Mobile exergames for GPS (global positioning system)-enabled smartphones and mini-tablets take players outdoors, in the open air, unlike console exergames, e.g., Xbox 360 Kinect exergames, which limit players to playing indoors in front of a TV set. In this paper and its companion ‘Additional file 1’, we review different examples of GPS exergames and of gamified geosocial apps and gadgets (mobile, location-aware apps and devices with social and gamification features), and briefly discuss some of the issues surrounding their use. Further research is needed to document best practices in this area, quantify the exact health and fitness benefits of GPS exergames and apps (under different settings and scenarios), and find out what is needed to improve them and the best ways to promote their adoption by the public.

## Background

Current guidelines for physical activity recommend that children and young people accumulate at least 60 minutes of moderate to vigorous physical activity (MVPA) daily. Unfortunately, very many children and adolescents aged 5–18 in Canada, UK and USA are not getting their recommended daily dose of MVPA [1-3], and are thus more prone to obesity and its ill health effects. Furthermore, research shows that children and adolescents are increasingly spending more time being sedentary while consuming various types of electronic and online media [4]. Physical inactivity (often associated with poor diet and overweight) has been blamed as one of the leading causes of death in the United States [5].

The problem is that, other than organised sports, there are not many enjoyable alternatives to obtain MVPA. However, exergames (video games that require physical activity or moving one’s body to play [6]) and GPS (global positioning system) exergames (electronic, location-based games that are played outdoors with the help of GPS in mobile devices carried by players) are rapidly gaining acceptance, and may have the potential to increase physical activity levels among young people.

Children and adolescents like playing video games [4]; but until recently, many did not see the use of video games as being beneficial in promoting physical activity. Challenging that belief is a group of newer console video games and controllers such as the Xbox Kinect [6,7], that allow players to physically interact with the game and burn more calories in the process (exergames). However, the biggest limitation of console exergames is that they require a television set or computer monitor to play, and cannot be played outdoors on a mobile device.

The adoption rate of smartphones has “exploded” in recent years and is said to be growing faster than that of any other consumer technology in history [8,9]. Among the most popular categories of smartphone apps are those dealing with health and fitness, exercise routines and dietary food intake, as well as social apps and games. Many smartphones and mini-tablets also incorporate GPS [10], and apps are now available that have capitalised on GPS and ‘network location’ functionalities in mobile devices, and incorporated game mechanics or gamification features that engage the user beyond the basic GPS functions of location, elevation, time, distance and speed, e.g., in alternate-reality games (ARG) and in other types of location-based exergames that require players to move and perform game tasks in a specific geographical area or in a non-pre-defined area. In this paper, only those mobile apps that have “advanced” GPS functions and game mechanics will be considered.

## Survey of GPS exergames and gamified geosocial apps and gadgets

**Figure 1:**
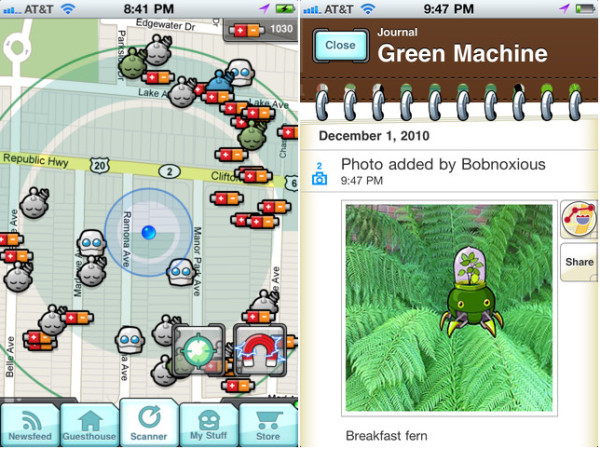
**Dokobots exergame for the iPhone/iPod touch/iPad.** Please refer to ‘Additional file 1’ for a detailed description of this product.

**Figure 2:**
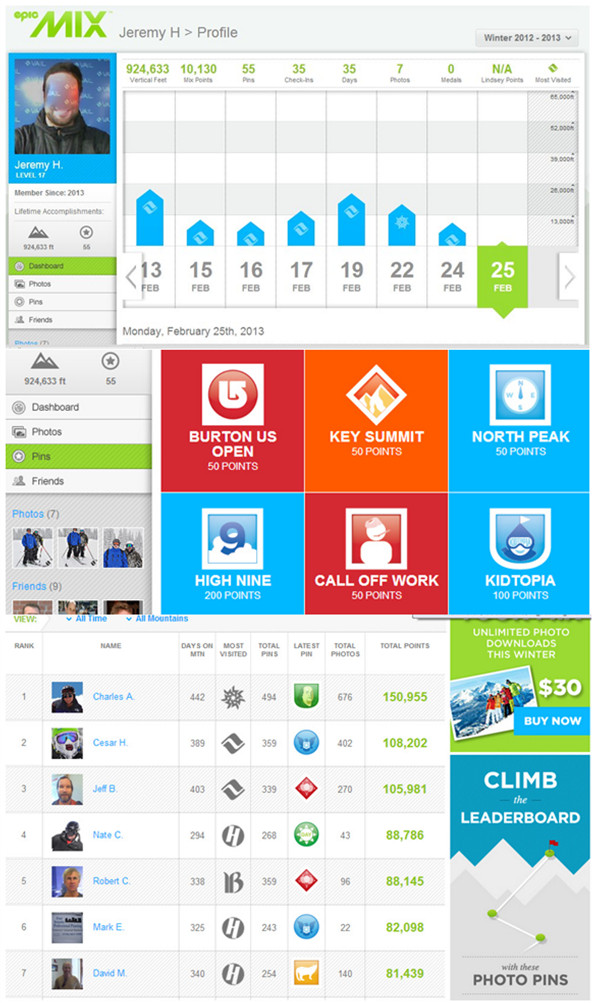
**Gamification features in EpicMix at Vail Resorts: pins (achievements) and leaderboards.** The geolocation technology used in EpicMix is RFID (radio-frequency identification) rather than GPS. Please refer to ‘Additional file 1’ for a detailed description of this product.

**Figure 3:**
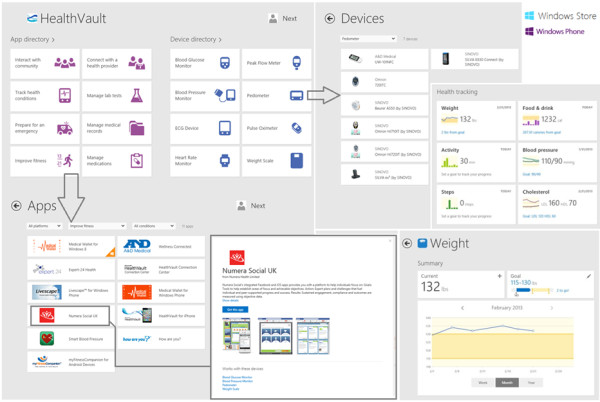
**Microsoft HealthVault for Windows 8/RT and Windows Phone.** Please refer to ‘Additional file 1’ for a detailed description of this product.

**Figure 4:**
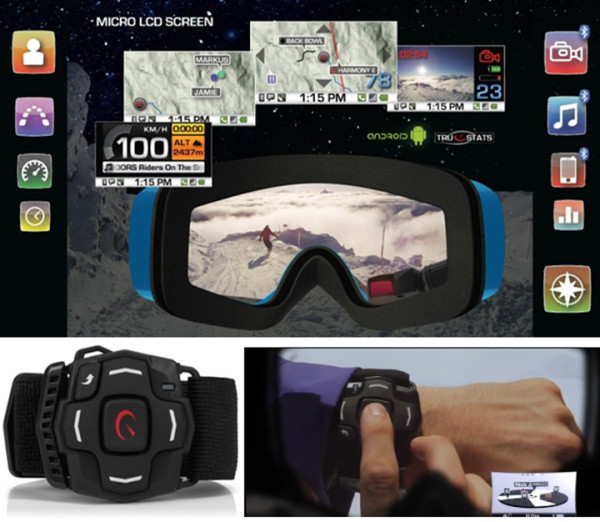
**Oakley GPS Goggle.** Please refer to ‘Additional file 1’ for a detailed description of this product.

We conducted an extensive online search of existing product literature about GPS exergames and geosocial apps and gadgets. We also scanned the most popular online mobile stores/marketplaces (e.g., Google Play [11]) for such apps. Our results are presented in ‘Additional file 1’. They cover: (i) mobile, location-based game apps, e.g., Dokobots (Figure 1); (ii) mobile, location-aware fitness and sports hubs/platforms with social and gamification features, e.g., EpicMix ski app (Figure 2) and Microsoft HealthVault (Figure 3); and (iii) location-aware sports gadgets, e.g., Oakley Airwave GPS-enabled Goggle for skiing (Figure 4) and products combining GPS and heart rate monitoring of the player.

## Discussion

Smartphone and GPS technologies are already playing important roles in telehealthcare and in medical emergencies [12-14], but here we are presenting some additional, non-conventional (in the context of health and healthcare) uses of these technologies, which can help keep our populations healthy and fit through the use of GPS in interesting and motivating outdoor games.

### Central themes of location-based exergames, game maps and user customisation options

The exergames presented in ‘Additional file 1’ cover a range of themes, including geocaching, (virtual) treasure hunting and item collection, trying to beat opponents and rank highest on leaderboards in multiplayer games, and games fashioned around visiting new locations, as in geodashing, geohashing, waymarking, etc. The latter group of exergames may also offer an educational opportunity to discover new places and learn about their geography and environment.

Bunker Buster [15], one of the geosocial games in our survey, uses Foursquare [16] venues as the game board. According to a study by the Pew Internet & American Life Project published in 2012, 18% of US smartphone owners over 18 used geosocial check-in apps such as Foursquare to “check in” to different locations and/or share their location with friends [17].

In location-based exergames, game maps (or playgrounds) are existing areas and streets in the real world outdoors. These maps and any associated virtual (or real) items on them, which players are supposed to avoid or find/collect or interact with in some other way, can be auto-generated (by the game engine/server) and/or user-generated (in those games that offer the option for players to create their own game maps). A good example of the latter is the Mission Designer mode of GPS Mission Pro (a GPS exergame), where anyone can create new missions for whatever location they have decided to use, and add game elements such as clues, photo tasks and bonus items [18].

Another example of user customisation and user-generated content offered by products in our survey is MovesCount App Zone, Suunto’s Ambit GPS watches companion app community and apps [19]. These sports apps, although not proper games as such, enable users to customise their GPS watches and to interactively define the parameters of their workout without any programming tools or knowledge. Using Suunto’s App Designer, users can even create (and share) their own apps, if they cannot find an existing app that satisfies their requirements.

### Shortcomings of GPS exergames

GPS technology issues are well documented elsewhere, e.g., in [12], and include location privacy issues and the GPS receivers sometimes failing to find a ‘fix’, e.g., when operating in some urban canyons, in heavy foliage environments, or in covered places, such as inside a large shopping mall or underground train station.

Although conquering new lands and getting new items is exciting no matter whom the player is battling or what he/she needs to collect, the biggest risk of auto-generated tokens, awards and enemies in GPS exergames is the safety of the game player. Players of GPS exergames can find themselves wandering in hazardous or prohibited/private areas if they do not exert due caution. The game might place an award in a dangerous location or it may be inaccessible by foot, and these compromising positions are a risk. In 2011, a mysterious object (black box) used in a GPS-based treasure hunt game caused a scare and resulted in parts of the Downtown Disney Park in Anaheim, CA, USA, to be temporarily closed down for a short time by the bomb squad [20].

GPS exergames, like other smartphone apps, can also be a potential source of malware [21], and users should only download and install apps from trusted sources, paying particular attention to the permissions they grant to each app.

Apps should ideally establish and adjust to individual players’ fitness profiles, warning users against playing for prolonged periods of time without taking enough rest (and hydration) and against engaging in dangerous exercise levels, particularly for those players with some pre-existing health condition, e.g., heart disease or type I diabetes (hypoglycaemia risk), or players with overall low fitness levels (when heavy exercise they are not previously accustomed to is suddenly introduced).

### The social/multiplayer effect

Many of the mobile exergames listed in ‘Additional file 1’ feature a prominent social or multiplayer component. In a recent study about console exergames, O’Donovan *et al.* found that playing the games in multiplayer mode led to greater energy expenditure and heart rate than playing in single player mode [22]. The same ‘multiplayer effect’ can be rather safely assumed to apply to mobile GPS exergames, but research is still needed to confirm (and quantify) this effect in outdoor settings.

### An SDT (self-determination theory)-based framework for future research

**Figure 5:**
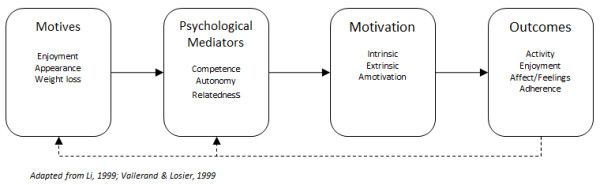
Relationships between SDT mediators, motivation, outcomes and motives.

To further improve location-based exergames and their uptake, more research is warranted and should focus on what youngsters like to do. Researchers should investigate games and activities that youngsters are already intrinsically motivated to play. The foundations for self-determination theory (SDT) can help parents, educators and exergame designers foster environments that develop self-determined motivation. To examine youngsters’ motives for participating in games they enjoy, the framework in Figure 5 was adapted from previous research on SDT and social-cognitive theory [23-25].

This framework suggests that individuals may have motives to participate in an activity, which may include enjoyment or physical appearance/weight loss. An example would be a girl who uses Nike + GPS Tag [26] to have fun and exercise. The motives to play Nike + GPS Tag feed into the mediators of the activity, which include competence, autonomy and relatedness. If the girl thinks that by playing Nike + GPS Tag she will feel competent, she will likely continue playing. Continued feelings of competence would likely lead to self-determined (intrinsically motivated) behaviour. Some of the outcomes from playing Nike + GPS Tag might include physical activity, enjoyment and adherence. Most of the available literature on SDT does not use a representational model like the one adapted in Figure 5. From a practical viewpoint, there should be some form of feedback from the experience of playing a game. In Figure 5, the feedback (arrows) from the outcomes, flow back into the motives and mediators. It makes more sense for the relationships to be cyclical as opposed to linear.

Parents, educators and community leaders need to foster socially supportive and fun environments for positive youth development. Research has shown that young people learn best when they are interested and engaged. Youngsters feel motivated when they are with their peers in voluntary settings, such as sports and hobbies. Youngsters then have a ‘window of opportunity’ to engage in activities in which they freely chose to participate. By empowering youngsters to choose for themselves, sports and physical activity (including activity arising from playing exergames) can provide unique environments that help build competence, autonomy and relatedness. Studies indicate that participation in voluntary structured activities during non-school time is associated with the development of positive identity, increased initiative and positive relationships with diverse peers and adults, better school achievement, reduced rates of dropping out of school, reduced delinquency and more positive outcomes in adulthood [27]. Location-based exergames that are played outdoors can potentially play an important role in all these aspects of youth development.

## Conclusion

Location-based exergames are here to stay. While many would prefer to see young people obtaining their physical activity through sports, it seems increasingly clear that for some youngsters growing up in the 21st century, GPS exergames may be a popular alternative (or supplement) to conventional sports such as football and skiing. Some of the apps and gadgets surveyed in this paper, such as EpicMix ski app, add additional dimensions to conventional sports. If GPS exergames can be as motivating to play as traditional video games, and they have the additional physical activity benefits, we should continue to research, explore and encourage their use. The ‘Game ON (Outdoor Nation)! Challenge Grants’ seem a good step in this direction [28]. Individuals and teams, beginners and experts alike, are invited to create a new app or mobile game, or promote an existing game, to inspire youth to get outdoors and active. Grants of US $5,000 will be given to the top four projects and the winners will also have the opportunity to attend the E3 video game conference and show in Los Angeles in June 2013.

## Competing interests

The authors declare that they have no competing interests.

## Authors’ contributions

MNKB conceived the manuscript’s idea and drafted the paper and companion ‘Additional file 1’, with professional physical education insight and contributions from SPY (ExerGame Lab). Both authors read and approved the final manuscript.

## Supplementary Material

Additional file 1 **List of GPS exergames and geosocial apps and devices.** PDF (Portable Document Format) document listing a large number of GPS exergames and geosocial apps/devices arranged in alphabetical order by game/app/gadget name, with short descriptions and Internet links. Click here for file
