# Supplementary material for: Exergames for health and fitness: the roles of GPS and geosocial apps
Source: Int J Health Geogr. 2013 Apr 5;12:18. doi: 10.1186/1476-072X-12-18 (PMC3657542; doi:10.1186/1476-072X-12-18)
Supplement: Additional file 1 — List of GPS exergames and geosocial apps and devices. PDF (Portable Document Format) document listing a large number of GPS exergames and geosocial apps/devices arranged in alphabetical order by game/app/gadget name, with short descriptions and Internet links. [file 1476-072X-12-18-S1.pdf]

GPS exergames and geosocial apps and devices (in alphabetical order by game/app/device name).

Game/app and/or device/gadget	Short description	Internet link(s)
CodeRunner	CodeRunner is a location-based (via GPS), iPhone/iPad espionage adventure exergame (*cf.* Google Ingress). The real world is the game map, and the story adapts to use locations near the player, so works anywhere on Earth. The game revolves around the player in the role of a secret agent, finding clues and solving mysteries of espionage in real-world streets. The gameplay feels genuine as it incorporates real-time GPS maps, with short videos of simulated characters involved with the player's story and his/her mission to find new evidence and also create new 'evidence drops' for others to play.	http://www.coderunnergame.com/<br>iPhone/iPad app: https://itunes.apple.com/app/coderunner/id463639902
***Coke Zero LiveCycle* app**	LiveCycle is a real-time, location-based outdoor video game with artificially-intelligent opponents, a multiplayer mode and Facebook sharing. The game offers a real-life version of Disney's TRON and makes use of the iPhone's accelerometer, compass and GPS to track player's movements. Available for iPhone/iPod/iPad.	https://itunes.apple.com/us/app/livecycle/id399611188?mt=8
Degree Confluence Project	Confluences are the points on Earth where longitude and latitude lines meet. The goal of this exergame project is for different players to visit each of the latitude and longitude integer degree intersections around the world, to take pictures at each location, and post these along with their visit stories on the project's website.	http://confluence.org/<br>United Kingdom example (click the different points on the map to access the corresponding stories and pictures): http://confluence.org/country.php?id=19
Dokobots	Dokobots is a geosocial exergame for the iPhone/iPod touch/iPad. Using an augmented map	http://www.dokobots.com/<br>iPhone/iPod/iPad app:

Game/app and/or device/gadget	Short description	Internet link(s)
	interface, players search for the hidden items in the game while roaming in the real world. Each player participates in a scavenger hunt to activate the highest number of Dokobots. Players collect inactive Dokobots and gems by my moving towards them. Once they encounter other Dokobots, players can take photos and share the images online (Figure 1).	https://itunes.apple.com/us/app/dokobots/id389267298
Endomondo Sports Tracker	GPS tracking of running, walking, cycling, etc., with a vibrant online community of >13 million users, and social features such as challenges (competitions with achievements and prizes), live peptalks from friends while exercising and Facebook sharing of workouts.	http://www.endomondo.com/<br>Challenges: http://www.endomondo.com/challenges/<br>Android app: https://play.google.com/store/apps/details?id=com.endomondo.android<br>Blackberry app: http://appworld.blackberry.com/webstore/content/7593/<br>iPhone/iPad app: https://itunes.apple.com/us/app/endomondo-sports-tracker-gps/id333210180?mt=8<br>Nokia Ovi Store app: http://store.ovi.com/content/3669<br>Windows Phone app: http://www.windowsphone.com/en-us/store/app/endomondo-sports-tracker/6cd31275-c5dd-df11-a844-00237de2db9e<br>Windows 8/RT app: http://apps.microsoft.com/windows/en-us/app/endomondo/6bccabc9-9515-4522-9b50-c66237088b0f/
EpicMix at Vail Resorts (Figure 2)	An RFID (Radio-Frequency IDentification)-based skiing/boarding social media platform with strong gamification features (online leaderboards and pins/achievements). EpicMix apps are available for Android and iPhone/iPod/iPad. Geolocation is achieved via RFID (in skiier's passes) rather than GPS.	http://www.epicmix.com/ - More details at: https://sites.google.com/a/utexas.edu/misepicmix2/development-of-epicmix and http://www.youtube.com/watch?v=b-uB94kab0I
Foursquare	Foursquare is a location-based social networking service for mobile devices with GPS hardware (can also use network location). It allows people to	https://foursquare.com/<br>*Turf Geography Club*: http://www.youtube.com/watch?v=nB_M6E3TNNc

Game/app and/or device/gadget	Short description	Internet link(s)
	share and save the places they visit. Users 'check in' at real-world venues, and with each check-in, they are awarded points and sometimes 'badges' or 'venue mayorships'. Foursquare had over 30 million users worldwide as of January 2013. Games are available that are based on Foursquare, e.g., *Turf Geography Club* (discontinued in February 2013), an iPhone/iPad Monopoly (board game) set on top of Foursquare.	
Geocaching apps/Travel bugs	Geocaches are hidden packages which are mapped for players to find. Inside the packages are useful bits and pieces that people have left behind, often with a book that the player can sign to say he/she has been there. Players are usually free to take something from the cache and replace it with something new for someone else to find.<br>A related exergame is played using travel bugs and geocoins. These are objects with barcodes and/or serial numbers attached to them, with some sort of instructions as to where the player wants them to go next. A player leaves a travel bug in a geocache; when a new player finds it, he/she reads the original player's instructions and takes the bug to the next logical cache. Bugs can be tracked via geocaching.com and similar sites to map the journeys that they take around the globe.	http://www.geocaching.com/<br>*GeoBeagle* (Android app):<br>https://play.google.com/store/apps/details?id=com.google.code.geobeagle<br>*Blackstar* (Blackberry app):<br>http://code.google.com/p/gpsnavigation/downloads/list<br>*Geocaching* (iPhone/iPod touch/iPad app):<br>https://itunes.apple.com/app/geocaching/id292242503?mt=8<br>Travel bugs can be bought online from:<br>http://www.geotastic.com/<br>http://www.aboveandbeyond.co.uk/travel%20bug/list/search
Geodashing	Geodashing is an exergame in which a number of random locations are selected for each game round, and the winner is the person or team who manages to visit the most locations before a set deadline.	http://geodashing.gpsgames.org/<br>Other GPS exergames at the parent site (gpsgames.org):<br>http://www.gpsgames.org/ - e.g., *Shutterspot*, a GPS exergame "in which some players take photographs and other players are challenged to find the exact spot where the photographer stood when the camera shutter clicked",

Game/app and/or device/gadget	Short description	Internet link(s)
		etc.
Geohashing apps	Geohashing is an adventure exergame comprising journeys to random places within a given area. There is a wiki of people geohashing worldwide.	http://wiki.xkcd.com/geohashing/<br>*Geohash Droid* (Android app): https://play.google.com/store/apps/details?id=net.exclaimindustries.geohashdroid<br>*GeoHashing* (iPhone/iPod touch/iPad app): http://itunes.apple.com/app/id522435211
GPS Mission Pro Ghost Patrol	GPS Mission Pro Ghost Patrol is a location-based ghost-hunting game where the player has to move towards the ghosts around him/her and use his/her weapons to defeat them and take their souls to power up and get new weapons. Players have to find and open safes by using the appropriate key to reveal new treasures as they try to defeat the Ghost King.<br>An online 'Mission Designer' mode of GPS Mission Pro is available where anyone can create new missions for whatever location they have decided to use, and add game elements such as clues, photo tasks and bonus items.	http://gpsmission.com/<br>GPS Mission Pro Mission Designer: http://gpsmission.com/missionadmin/create.do<br>Windows Mobile 5 and 6 app: http://gpsmission.com/Download-WinME-phones.htm<br>Samsung app (Java and Windows Mobile): http://gpsmission.com/Download-Samsung-phones.htm<br>Nokia app (Java): http://gpsmission.com/Download-Nokia-phones.htm<br>iPhone app: no longer available (archived version: http://web.archive.org/web/20100301040721/http://itunes.apple.com/app/gps-mission-pro/id327569371?mt=8)
***HealthVault* (Microsoft)**	HealthVault serves as a 'hub' for personal health and fitness tracking, apps (including social apps such as Numera Social) and devices such as weight scales, heart rate monitors and pedometers (Figure 3).	HealthVault for Windows 8/RT: http://apps.microsoft.com/windows/en-GB/app/healthvault/728f1c88-7e2f-4b40-95c1-74fc09983689<br>HealthVault for Windows Phone: http://www.windowsphone.com/en-gb/store/app/healthvault/daa5bf42-cf02-4327-97cf-0721ca4ff7a2
***Ingress* (Google)**	A location-based game (in closed-beta as of March 2013) that transforms the real world around the player into a landscape for a global game of mystery, intrigue and competition (*cf.* iPhone CodeRunner). In the game story, the future is at stake as an unknown energy source is changing the	http://www.ingress.com/ and http://www.nianticproject.com/<br>Android app: https://play.google.com/store/apps/details?id=com.nianticproject.ingress

Game/app and/or device/gadget	Short description	Internet link(s)
	way we think and players must take control of it before it controls them. Players have to find and acquire sources of this energy by capturing new territories in a 'good vs. evil' global battle. Unlike other apps that have smaller group-play and worlds, this game uses the entire world as its playing field and only two teams are battling for supremacy.	
***LocoMatrix* (multiple games)**	A range of smartphone games (single player or multiplayer) that are played outdoors based on the player's GPS position.	http://www.locomatrix.com/<br>*Invisible Buildings* (GPS game example for school kids): http://schools.locomatrix.com/
***MINI Getaway Stockholm 2010* (no longer available to play)**	An advergame (ran between 31 October - 7 November 2010) that challenged each player to hunt, catch and escape with a virtual MINI car in Stockholm using a GPS-powered iPhone app and the player's own two legs. The player had to get within 50m of the virtual MINI and hit 'Take the MINI Now' in the app before escaping as fast as possible, since anyone else who got within 50m of the player could instantly take the virtual MINI back. The person holding the virtual MINI after seven days of 24/7 gaming won a real MINI Countryman.	http://www.minigetawaystockholm.com/ and http://www.youtube.com/watch?v=WMWu1h_6OfE<br>Archived iPhone app page on iTunes: http://web.archive.org/web/20101023014554/http://itunes.apple.com/se/app/mini-getaway-stockholm/id396397148?mt=8
***Miscellaneous GPS-enabled Android outdoor game apps* (mostly arcade/action)**	See corresponding Internet links for Google Play (Android marketplace) descriptions.	*Android Hunt* (RPG [Role-Playing Game]-styled location-based [GPS] game) https://play.google.com/store/apps/details?id=com.wh.androidhunt<br>*Bunker Buster* (a geo-social game with Foursquare [https://foursquare.com/] venues as the game board): https://play.google.com/store/apps/details?id=com.projectzebra.bunkerbuster<br>*CYA Claim Your Area* (a GPS-powered active strategy game in which players move virtual droids, harvest areas and conquer fields on the global game

Game/app and/or device/gadget	Short description	Internet link(s)
		plan; Facebook-enabled)<br>https://play.google.com/store/apps/details?id=wOd.View<br>*Dig A What* (a location-based virtual treasure hunt)<br>https://play.google.com/store/apps/details?id=com.emistra.digawhat<br>*Fantasy GPS Wars*<br>https://play.google.com/store/apps/details?id=com.droidbd.fantasy<br>*Feudalsquare* (turns player's city into a medieval adventure; uses the foursquare® API—Application Programming Interface)<br>https://play.google.com/store/apps/details?id=com.feudalsquare.core<br>*FreshAiR* (augmented reality app and editor):<br>https://play.google.com/store/apps/details?id=com.mogomobile.freshair<br>*GPS Onslaught FREE* (multiplayer shooter/defence game)<br>https://play.google.com/store/apps/details?id=org.me.gpsonslaught<br>*GPS Earth Defense*<br>https://play.google.com/store/apps/details?id=com.zelfi.android.gpsEarthDefense<br>*MobileWar* (multiplayer GPS shooting game with the phone as the 'shooting device')<br>https://play.google.com/store/apps/details?id=com.mobilewar<br>*PokerWalk* (multiplayer: players race friends around their actual neighbourhood in search of virtual poker cards)<br>https://play.google.com/store/apps/details?id=com.urban_detection.poker_walk<br>*Rex Marks The Spot* (scavenger hunt with GPS and YouTube videos)<br>https://play.google.com/store/apps/details?id=com.zadov<br>*Seek 'n Spell* (players gather virtual letters by running around an outdoor space to win points by spelling words with collected letters)<br>https://play.google.com/store/apps/details?id=com.retronyms.android.seeknspell_heavy<br>*Skirmsh* (a war-themed, location-based [GPS] online RPG game)<br>https://play.google.com/store/apps/details?id=com.linle.skirmsh

Game/app and/or device/gadget	Short description	Internet link(s)
		Tourality GPS Treasure Hunt (single and multi-player outdoor GPS game; a location-based scavenger hunt where player collects gold with up to 40 other players in his/her area)<br>https://play.google.com/store/apps/details?id=com.tourality.client.android<br>*Virtual Orienteering* (inspired by orienteering sports)<br>https://play.google.com/store/apps/details?id=com.vorienteering.virtualorienteering<br>*Zombie Stickmen Invasion*<br>https://play.google.com/store/apps/details?id=com.wh.zombie<br>*Zombie Survival Runner*<br>https://play.google.com/store/apps/details?id=com.halffastapps.zombiesurvivalrunner<br>*Zombie Zurvival Zystem*<br>https://play.google.com/store/apps/details?id=com.aldertreecove.zombiezurvivalzystem
Mobile Adventure Walks	A treasure hunting exergame for the solo explorer as well as for groups. App asks player a question based on his/her location. The questions have to do with something the player can see immediately around him/her.	http://www.mobileadventurewalks.com/<br>iPhone/iPod touch/iPad app: https://itunes.apple.com/us/app/mobile-adventure-walks/id464283791?mt=8
Moves	Combines accelerometry and GPS for physical activity monitoring on the iPhone. Moves is 'always on', offering an automatic diary of user's life, with daily 'storyline' and maps to show where, when and how much the user has moved. Not a proper game yet, but could have great possibilities if its API (Application Programming Interface) is open.	http://moves-app.com/<br>iPhone/iPad app: https://itunes.apple.com/us/app/moves/id509204969?mt=8
Nike+ Running app	Makes use of smartphone's existing GPS and accelerometer for tracking running distance, pace, time and calories burned. Players can post the start of their run to Facebook and hear real-time cheers	http://nikeplus.nike.com/plus/products/gps_app/

Game/app and/or device/gadget	Short description	Internet link(s)
	for each 'like' or comment they receive. Available for Android and iPhone/iPod/iPad.	
***Oakley Airwave - GPS Enabled Goggle* (Figure 4)**	Similar to *ZEAL Optics Z3™ GPS Goggle* (see below), this ski smart-goggle comes with a built-in heads-up display perceived to be the size of a 14-inch screen viewed from a distance of five feet. Features GPS navigation, player analytics and buddy tracking, smartphone (iPhone or Android) Bluetooth connectivity and a music player.	http://oak.ly/YwNlmy<br>*cf. Google Glass*: http://www.youtube.com/watch?v=v1uyQZNg2vE
***Passage* (Aetna, Inc.)**	Passage transforms the outdoor running, walking and cycling that players track using FitBit or RunKeeper into a geographic exploration game-like experience. A kilometre recorded in RunKeeper or FitBit is a kilometre that the player progresses through Passage, unlocking beautiful photos, facts, quotes, etc. along virtual courses set through real world cities. This enables the player to virtually visit what he/she would see if they were actually travelling along these routes and walking the avenues of those cities.	Windows 8/Windows RT app: http://apps.microsoft.com/windows/en-us/app/passage/ebf9baed-a745-4430-b49d-60b3027d66de<br>FitBit: http://www.fitbit.com/<br>RunKeeper: http://runkeeper.com/
***Polarpersonaltrainer.com* (Polar Electro)**	An online service from Polar, where users can create sports training targets and follow their progress together with their friends. Users can challenge their friends and find new friends from Polarpersonaltrainer.com community to share their trainings with them. Works with Polar's GPS sports watches such as the RC3 GPS Heart Rate watch, which offers integrated GPS with heart rate monitoring.	https://www.polarpersonaltrainer.com/
***RunAway* (PlaceChallenge)**	RunAway is a GPS exergame where players run around the city trying to find, then collect and	http://www.runawayapp.com/<br>http://placechallenge.com/

Game/app and/or device/gadget	Short description	Internet link(s)
	protect a virtual token on their phone or tablet. The player who manages to keep the token till the end of the game wins. Once a player finds the token, he/she will have it on the phone or tablet, and should keep running around the city, avoiding other players in order to keep the token in his/her possession. Any other player who happens to be closer than 50 metres from the current token owner can grab the token to own phone/tablet and so forth. There is always one player running away, while others are going after him/her. Players can see each other on the map, so the target's position is always known.	Android app: https://play.google.com/store/apps/details?id=com.placechallenge.games.runaway_v1<br>iPhone app: http://www.runawayapp.com/iPhone<br>Windows Phone 7 app: http://www.windowsphone.com/en-us/store/app/runaway/74304bf7-a154-4394-9c3a-5f575dae4911<br>Windows 8/RT app: http://apps.microsoft.com/windows/en-us/app/RunAway/d8ea7ac5-d13d-42ef-8b82-6ba88173f60d
***Serpent* (PlaceChallenge)**	A GPS exergame for two people, that takes place outdoors. Players choose start positions and trace their own track, while keeping an eye on their friend's track, trying to cut him/her off to win the game.	http://serpentapp.com/<br>Android app: https://play.google.com/store/apps/details?id=com.placechallenge.games.serpent<br>iPhone app: http://serpentapp.com/iPhone<br>Windows Phone 7 app: http://www.windowsphone.com/en-us/store/app/serpent/68c7f1ef-b0e3-41b0-94af-c8991d0a3101<br>Windows 8/RT app: http://apps.microsoft.com/windows/en-us/app/Serpent/81f6917e-77cd-4b62-b681-e0198150f1c9
Suunto Movescount	An online sports community with apps compatible with Suunto's GPS-enabled devices, e.g., Suunto Ambit.	http://www.suunto.com/us/products/software/Movescount and http://www.movescount.com/apps<br>iPhone/iPad app: https://itunes.apple.com/us/app/movescount/id465054490
TrezrHunt	Developed by Team Action Zone (TAZ) in Finland, TrezrHunt can be played against one to five other people. In the game, a series of treasures are hidden around an area ranging from 100 to 1,000 square metres. To earn game points, players need to find these treasures with the aid of their GPS	http://www.trezrhunt.com/<br>Android app: https://play.google.com/store/apps/details?id=fi.taz.android.trezrhunt<br>Nokia Ovi store app: http://store.ovi.com/content/213687<br>More GPS exergames by TAZ: http://www.taz.fi/?page_id=203 and http://www.youtube.com/watch?v=fZzeGHkg5tQ

Game/app and/or device/gadget	Short description	Internet link(s)
	phones. However, points can be lost by bumping into a white ghost while moving around. If a player bumps into a red ghost, the game is over. Players can use traps to stop the ghosts in their tracks and earn some extra points. At the end, the player who has the most points wins. Other GPS exergames offered by the same company (TAZ) include ActionTrack and FlagHunt.	
Urban Dash	A GPS-powered advergame by New Balance Athletic Shoe, Inc., with daily-released virtual batons to hunt throughout New York City and real prizes to win.	https://itunes.apple.com/us/app/urban-dash/id452952594?mt=8
Waymarking	In waymarking, players share the co-ordinates and details of interesting locations in order to build a community map of cool places. Players can then take part in scavenger hunts using their smartphones and the waymarks near them.	http://www.waymarking.com/
ZEAL Optics Z3™ GPS Goggle	Multi-function ski smart-goggle with GPS and smartphone Bluetooth connectivity. An in-goggle view-finder displays all of the player's stats and friends' location on the mountain on a 16:9 widescreen. Controls for the goggle are managed through a Bluetooth-enabled remote that the player wears on the wrist like a watch.	http://www.zealoptics.com/z3.html and http://www.youtube.com/watch?v=F9u1mUlK8qg<br>*cf. Google Glass*: http://www.youtube.com/watch?v=v1uyQZNg2vE
***Zombie, Run!* and *5k Training* apps**	GPS-enabled, immersive running games and audio adventures with 300,000+ players worldwide. Players can view their run logs and statistics, including calories burned and zombies evaded, and share logs with friends on Facebook and Twitter. Available for Android, iPhone/iPod/iPad and Windows Phone.	https://www.zombiesrungame.com/

N.B.: For Figures 1-4, please refer to the main article entitled 'Exergames for health and fitness: the roles of GPS and geosocial apps' in *International Journal of Health Geographics*, Volume 12 (2013).
